# Crossmodal effect of music and odor pleasantness on olfactory quality perception

**DOI:** 10.3389/fpsyg.2014.01352

**Published:** 2014-11-28

**Authors:** Carlos Velasco, Diana Balboa, Fernando Marmolejo-Ramos, Charles Spence

**Affiliations:** ^1^Crossmodal Research Laboratory, Department of Experimental Psychology, University of Oxford, Oxford, Oxfordshire, UK; ^2^School of Psychology, Faculty of Health Sciences, University of Adelaide, Adelaide, SA, Australia

**Keywords:** crossmodal correspondences, olfaction, audition, pleasantness, white noise

## Abstract

Previous research has demonstrated that ratings of the perceived pleasantness and quality of odors can be modulated by auditory stimuli presented at around the same time. Here, we extend these results by assessing whether the hedonic congruence between odor and sound stimuli can modulate the perception of odor intensity, pleasantness, and quality in untrained participants. Unexpectedly, our results reveal that broadband white noise, which was rated as unpleasant in a follow-up experiment, actually had a more pronounced effect on participants’ odor ratings than either the consonant or dissonant musical selections. In particular, participants rated the six smells used as being less pleasant and less sweet when they happened to be listening to white noise, as compared to any one of the other music conditions. What is more, these results also add evidence to support the existence of a close relationship between an odor’s hedonic character and the perception of odor quality. So, for example, independent of the sound condition, pleasant odors were rated as sweeter, less dry, and brighter than the unpleasant odors. These results are discussed in terms of their implications for the understanding of crossmodal correspondences between olfactory and auditory stimuli.

## INTRODUCTION

Researchers have demonstrated that olfactory perception can be influenced by inputs from the other senses presented prior to and/or at the same time (see Calvert et al., 2004; Stein, 2012 for reviews). In fact, a variety of crossmodal correspondences—the name given to the tendency for people to match the information presented in one sensory modality to that presented in another (see Spence, 2011, for a review)—have been documented between olfaction and the other senses (e.g., Stevenson et al., 2012; Deroy et al., 2013). So, for example, to date, crossmodal associations have been demonstrated between odors and pitch (Belkin et al., 1997; Deroy et al., 2013), brightness (Kemp and Gilbert, 1997), colors (e.g., Schifferstein and Tanudjaja, 2004; Demattè et al., 2006a; Maric and Jacquot, 2013), basic tastes (Urdapilleta et al., 2006), and even tactile stimuli (Demattè et al., 2006b).

Deroy et al. (2013) recently suggested that people may use descriptors or attributes that are normally associated with other sensory modalities such as pitch, brightness, and sweetness to describe olfactory stimuli/percepts because of the various crossmodal correspondences that exist between the senses. It is interesting to note, though, that the crossmodal interactions that link olfactory and auditory stimuli have received far less attention than, for example, those that exist between vision and audition (Seo and Hummel, 2011). Nonetheless, over the last couple of decades, researchers have started to document the existence of a number of crossmodal correspondences between olfactory and auditory stimuli. For instance, Belkin et al. (1997) provided one of the first examples of olfactory–auditory correspondences, showing that people would match a series of odors varying in quality, to sounds that differed in terms of their pitch (cf. Piesse, 1891). These results were later extended by Crisinel and Spence (2012) who also found that people tended to match certain odors to the timbres of particular musical instruments (see also Crisinel et al., 2013).

While the crossmodal correspondence between specific olfactory stimuli and, say, particular colors could be explained by their co-occurrence in foodstuffs (think only of the red color of strawberries and the associated aroma), it is far harder to think of an environmental explanation that could explain why people would reliably (or consistently) associate specific musical parameters with particular olfactory stimuli and why these associations would, in turn, influence information processing (see Belkin et al., 1997; Deroy et al., 2013). In their recent review, Deroy et al. (2013) suggested that one potential explanation for the existence of crossmodal correspondences between auditory and olfactory stimuli may sometimes be related to the hedonic properties of the stimuli presented in the two modalities (see also Stevenson et al., 2012). As Marks (1978, p. 181) indicated, sensory qualities “*talk over their common feeling*.” This notion has also been supported by recent research pointing to the idea that the emotional (hedonic) similarity between olfactory and auditory information may be crucial to both crossmodal correspondences and multisensory information processing (see Seo and Hummel, 2011; Crisinel and Spence, 2012; Hanson-Vaux et al., 2013).

Seo and Hummel (2011) reported two experiments in which they assessed the effect of auditory stimulation on the perceived pleasantness and intensity of odors. In their first study, they investigated whether auditory cues that were semantically congruent with the olfactory stimuli (sounds that were crossmodally better matched to the odors, i.e., the smell of potato chips while listening to the sound of crunching crisps) would modulate the perceived intensity and pleasantness of the odor. In their second experiment, the authors evaluated whether odor intensity and pleasantness could be influenced simply by manipulating the hedonic valence of the auditory stimuli.

Seo and Hummel (2011) reported that olfactory stimuli (such as the smell of coffee) were rated as more pleasant when paired with congruent sounds (i.e., when listening to the sound of drinking coffee in this example) as compared to incongruent sounds, and that the hedonic valence associated with the sounds can be transferred crossmodally, and thus influence people’s odor evaluations. Interestingly, however, no crossmodal effect on the perceived intensity of the odors was reported. Seo and Hummel (2011) suggested that the results of their first study may have been attributable to the existence of a crossmodal congruency effect (e.g., Shams and Seitz, 2008), while the results of their second experiment could be explained in terms of some form of halo/horn effect (e.g., see Thorndike, 1920; Kappes et al., 2006). This evidence points to a hedonic similarity account. It may be the case that there is a crossmodal transfer of the hedonic evaluation of the information presented in one sensory modality onto the information processed in the other.

Seo et al. (2014) recently extended Seo and Hummel’s (2011) results to more abstract auditory cues. In three experiments, the authors demonstrated that people match a variety of odors and background music, that congruent (to odors) background music (i.e., Christmas carols) would enhance the rated pleasantness of certain odors, and that congruent sounds had an impact upon odor familiarity and identification. For example, participants liked the smell of cinnamon more when it was presented together with (congruent) Christmas carols as compared to incongruent sounds. The authors suggested that the association between cinnamon and Christmas is very typical for the German participants whom they tested. The statistical regularities of the environment and co-occurrence of multisensory stimuli can thus lead to specific crossmodal associations (Shams and Seitz, 2008).

Further research is still needed in order to clarify the potential influence of auditory cues in odor perception, and its underlying mechanisms, since the crossmodal interactions between these senses are likely to operate at various different levels of human information processing (see Deroy et al., 2013). In the present study, we aimed to assess any effect of the pleasantness of the music on the perceived pleasantness and/or intensity of the odors. In addition, we also wanted to assess whether having congruent (vs. incongruent, in terms of the perceived pleasantness) auditory and olfactory stimuli would influence the perception of other odor qualities such as sweetness, brightness, acidity, and dryness, which are attributes commonly used to describe fragrances (e.g., in the context of perfumery; Zarzo and Stanton, 2009) and have been shown to be crossmodally associated to odors (Stevenson et al., 2012).

In the present study, we manipulated the crossmodal congruency based on a matching of the hedonic qualities of the stimuli. There is an intriguing question here as to whether and why changing what one hears can change an observer’s experience of an olfactory stimulus. There is a need to clarify both the nature of the specific associations that exist between odors and sounds, and the influence that auditory cues may exert on the perception of odor attributes in, for example, the case of fine fragrances.

## MATERIALS AND METHODS

### PARTICIPANTS

Thirty-three untrained participants (20 females, *M*_age_ = 31.9 years, SD_age_ = 10.65, range = 18–58 years) took part in this experiment, which was approved by the Central University Research Ethics Committee of the University of Oxford. The experiment was conducted in accordance with the ethical guidelines laid down by the Department of Experimental Psychology, at the University of Oxford.

The participants gave their informed consent and completed two questionnaires in order to assess any potential sensory dysfunction prior to their taking part in the study: an adapted version of the Hearing Handicap Inventory for the Elderly (HHIE-S) for auditory functioning (Ventry and Weinstein, 1983) and the Multi-Clinic Smell and Taste Questionnaire (MCSTQ-Sc, olfactory section) for olfactory dysfunction (see Nordin et al., 2003) were used, and only those participants who did not report any dysfunction were included. The experiment lasted for approximately 40–50 min. The participants were compensated for their time with £8.

### APPARATUS AND MATERIALS

Six odors, one from Le Nez du Café (Brizard and Co, Dorchester, UK) and five from *Le Nez du Vin* kits (Brizard and Co, Dorchester, UK), were selected as the olfactory stimuli. These kits have been designed to help those who would like to learn more about coffee/wine to experience a number of the characteristic odors commonly found in the different kinds of coffee/wine. In addition, they have been previously used in research involving crossmodal associations to odors (e.g., Crisinel and Spence, 2012; Hanson-Vaux et al., 2013). The samples were selected based on a preliminary experiment (see below) and consisted of odors identified as lemon, orange, and bilberry (all rated as pleasant), and musk, dark chocolate, and smoked (all rated as unpleasant).

**FIGURE 1:**
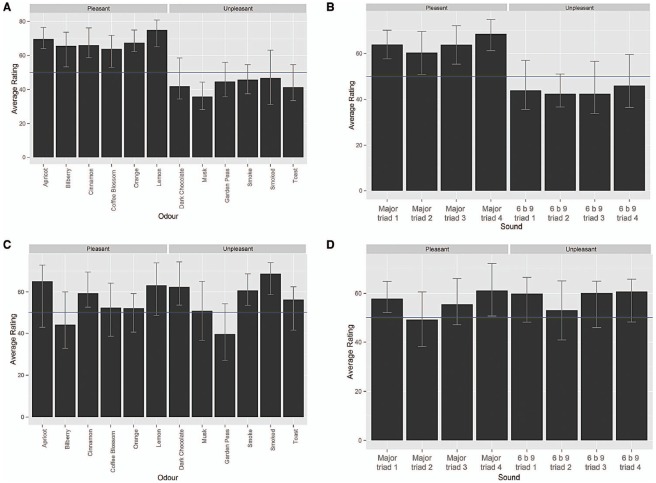
**Participants’ average (Harrell–Davis estimator of the median; Vélez and Correa, 2014) pleasantness ratings for the odors (A) and for the sounds (B), and intensity ratings for the odors (C) and sounds (D).** The ratings were performed on a scale ranging from 0 (not at all) to 100 (very). The error bars represent 95% bias-corrected-and-accelerated bootstrap confidence intervals.

In the preliminary study, 12 participants (seven females and five males, *M*_age_ = 24.9 years, SD_age_ = 5.7 range = 19–40) rated both the pleasantness and intensity of a variety of odors from Le Nez du Café (Coffee Blossom, Garden Peas, and Smoke) and Le Nez du Vin (Apricot, Bilberry, Cinnamon, Dark Chocolate, Orange, Lemon, Musk, Smoked, and Toast) kits. The ratings were made on visual analog scales (VAS) anchored between *not at all* (0) and *very* (100), which were presented using the E-Prime 2.0 software (Psychology Software Tools, Inc.). A summary of the results for the perceived pleasantness and intensity ratings are shown in Figures 1A,C, respectively. It is important to note here that, although it may seem surprising to find that the participants found the smell of dark chocolate to be unpleasant, previous research has also documented that certain odors (e.g., parmesan cheese, truffle, and pepper, see Seo et al., 2010), when presented out of (a food-related) context, can be perceived as unpleasant (Deroy et al., 2013).

The selected odors were those that were given the most extreme hedonic ratings (in terms of either their pleasantness or unpleasantness). The intensity ratings of the selected odors were analyzed by means of a repeated measures analysis of variance (ANOVA) in order to determine whether participants perceived the intensity of the odors differently. The ANOVA did not reveal any statistically significant difference between the ratings.

In addition to the selected odors, a no-odor (baseline) condition was also included, which consisted of participants sniffing an odorless empty bottle. All of the samples were presented in small glass bottles, covered with opaque paper, and hidden in black containers in order to prevent the participants from seeing the color of the sample liquids (cf. Crisinel and Spence, 2012).

The auditory stimuli were created with the software Finale (Finale, 2012; MakeMusic^®^, Inc.). They consisted of three musical fragments played on the piano with the very same melodies but different, in terms of their dissonance (i.e., pleasantness), triad chords accompaniments. The dissonance of the triads went from the most consonant fragment (major triad) to the most dissonant fragment (6 ♭9 triad), giving rise to six musical fragments (three consonant, three dissonant). These musical fragments can be heard at https://soundcloud.com/xmodal/sets/sounds-odours. The sounds were designed on the basis of previous research suggesting the existence of an association between different dissonant and consonant musical pieces and pleasantness/unpleasantness (see Blood et al., 1999; Sammler et al., 2007). White noise (70 dB) was used as a putatively neutral stimulus. The sounds were edited in Audacity (Audacity^®^, the Free, Cross-Platform Sound Editor) to last for exactly 10 s, and were presented over headphones (Sony MDR-NC6 Noise Canceling) at a loudness level of 70 dB.

The consonant and dissonant auditory stimuli were assessed in a preliminary study in order to determine whether they were rated as expected; either pleasant (consonant) or unpleasant (dissonant). The same participants who had taken part in the odor pre-test also took part in this pre-test (the order was counterbalanced). The participants rated the pleasantness of the odors using a VAS (from 0 = not at all, to 100 = very). The mean pleasantness and intensity ratings are highlighted in Figures 1B,D, respectively. The participants rated each of the musical pieces as either pleasant or unpleasant as expected.

### PROCEDURE

A within-participants experimental design was utilized with two factors: music (pleasant, unpleasant, and white noise) and odor (pleasant, unpleasant, and no-odor). The participants sat approximately 60 cm from a computer (with a screen resolution of 1366 × 768 pixels, and a screen refresh rate of 60 Hz), in order to perform the task. The instructions and response scales were presented on the computer monitor, and were also presented verbally in order to avoid any confusion.

Forty-nine different combinations of olfactory and auditory cues (seven odors and seven sounds, including the no-odor and white noise conditions) were presented in a random order to the participants. The auditory stimuli were presented for 10 s. Approximately 5 s after the onset of the auditory stimuli, the odor was presented, for the next 5 s, under the participant’s nose by the experimenter at the same moment as an instruction on the screen said *Please sniff the odor once*. The participants were asked to sniff the odor just once in order to control that the total number of sniffs per session was the same across participants.

After the presentation of the stimuli, the participants had to rate a number of odor attributes (pleasantness, intensity, sweetness, brightness, acidity, and dryness) on VASs anchored between *not at all* and *very much*. Note that participants were not instructed about how they should interpret the different attributes included. However, some of these are commonly used attributes when describing fragrances (e.g., in the context of perfumery; Zarzo and Stanton, 2009) or have been shown, in the olfactory domain, to be crossmodally matched to odors (Stevenson et al., 2012). What is more, there has been growing interest in odors taking on gustatory properties such as sweetness or sourness (Stevenson and Boakes, 2004), and dryness (Zarzo and Stanton, 2009), while brightness has been shown to be crossmodally associated to odors of different qualities (see von Hornbostel, 1931; Cohen, 1934; Kemp and Gilbert, 1994, 1997). To minimize any olfactory desensitization, there was a 15 s gap between the odor presentations, which together with the time participants took to respond to the scales made approximately 25 s between odor presentations. White noise was presented during this time (cf. Seo and Hummel, 2011).

## RESULTS

**FIGURE 2:**
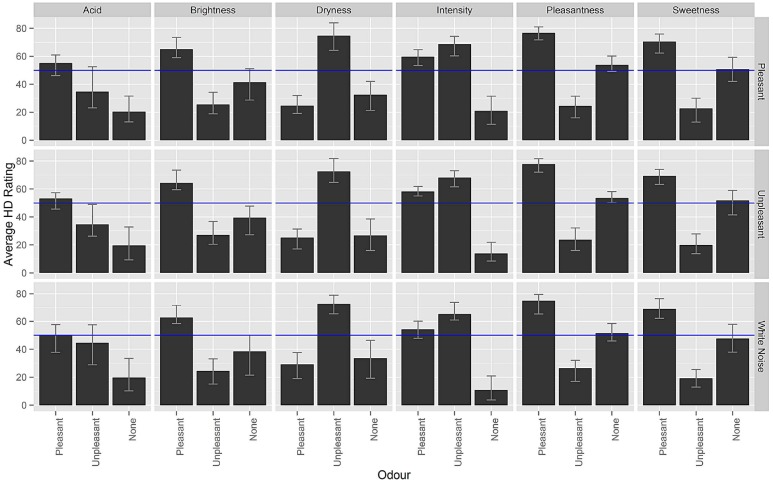
**Average ratings (Harrell–Davis estimator of the median) for each attribute in each odor and musical condition.** The error bars represent 95% bias-corrected-and-accelerated bootstrap confidence intervals (Efron, 1987).

Average ratings obtained by participants for each odor–music combination were estimated using the Harrell–Davis estimator of the median (Vélez and Correa, 2014). Note that a number of assumptions for parametric tests were violated (e.g., the data was not normally distributed), thus, a robust analysis, the rank-based ANOVA-type statistic^1^ (see Noguchi et al., 2012), was used to analyze the results. Average ratings were submitted to this ANOVA with the factors of music (pleasant, unpleasant, or white noise), and odor (pleasant, unpleasant, or odorless) for each of the odor attributes that the participants had to rate (pleasantness, intensity, sweetness, brightness, acidity, and dryness). Figure 2 displays the average ratings obtained and Table 1 shows the results of the analyses.

**Table 1:** Results of the ATS performed on each of the attributes assessed in the experiment.

	**ANOVA-type statistic analyses and two-sample permutation tests**		
**Odor attributes**	**Main effects**	**Interactions**	**Pairwise comparisons (M_HD_) [*p*-value_FDR_]**
Pleasantness	M: *F*_(1.69,∞)_ = 4.41, *p* = 0.016	M × O: *F*_(2.81,∞)_ = 0.74, *p* = 0.519 (ns)	M: WN (50.23) vs. PM (54.04): *p* = 0.18 [0.33]
	O: *F*_(1.87,∞)_ = 89.28, *p* < 0.001		O: WN (50.23) vs. UM (53.42): *p* = 0.22 [0.33]
			PM (54.04) vs. UM (53.42): *p* = 0.41 [0.41]
			NO (52.69) vs. PO (76.74): *p* < 0.001 [0.001]
			NO (52.69) vs. UO (25.50): *p* < 0.001 [0.001]
			PO (76.74) vs. UO (25.50): *p* < 0.001 [0.001]
Intensity	M: *F*_(1.77,∞)_ = 1.07, *p* = 0.33	M × O: *F*_(3.57,∞)_ = 1.15, *p* = 0.32 (ns)	O: NO (13.96) vs. PO (57.61): *p* < 0.001 [0.001]
	O: *F*_(1.53,∞)_ = 81.94, *p* < 0.001		NO (13.96) vs. UO (67.54): *p* < 0.001 [0.001]
			PO (57.61) vs. UO (67.54): *p* < 0.001 [0.001]
Sweetness	M: *F*_(1.83,∞)_ = 3.16, *p* = 0.046	M × O: *F*_(2.85,∞)_ = 1.12, *p* = 0.33 (ns)	M: WN (44.21) vs. PM (47.61): *p* = 0.28 [0.42]
	O: *F*_(1.81,∞)_ = 57.12, *p* < 0.001		O: WN (44.21) vs. UM (47.90): *p* = 0.22 [0.42]
			PM (47.61) vs. UM (47.90): *p* = 0.51 [0.51]
			NO (50.14) vs. PO (69.65): *p* < 0.001 [0.001]
			NO (50.14) vs. UO (20.55): *p* < 0.001 [0.001]
			PO (69.65) vs. UO (20.55): *p* < 0.001 [0.001]
Dryness	M: *F*_(1.68,__)_ = 3.70, *p* = 0.031	M × O: *F*_(1.76,__)_ = 1.05, *p* = 0.34 (ns)	M: WN (42.85) vs. PM (41.06): *p* = 0.36 [0.79]
	O: *F*_(1.71,∞)_ = 63.75, *p* < 0.001		O: WN (42.85) vs. UM (38.46): *p* = 0.79 [0.79]
			PM (41.06) vs. UM (38.46): *p* = 0.68 [0.79]
			NO (31.34) vs. PO (25.71): *p* = 0.89 [0.89]
			NO (31.34) vs. UO (73.14): *p* < 0.001 [0.001]
			PO (25.71) vs. UO (73.14): *p* < 0.001 [0.001]
Acidity	M: *F*_(1.97,∞)_ = 0.74, *p* = 0.47	M × O: *F*_(2.89,∞)_ = 2.29, *p* = 0.07 (ns)	O: NO (19.61) vs. PO (53.80): *p* < 0.001 [0.001]
	O: *F*_(1.52,∞)_ = 11.27, *p* < 0.001		NO (19.61) vs. UO (36.85): *p* < 0.001 [0.001]
			PO (53.80) vs. UO (36.85): *p* < 0.001 [0.001]
Brightness	M: *F*_(1.94,∞)_ = 1.56, *p* = 0.21	M × O: *F*_(2.30,∞)_ = 0.33, *p* = 0.74 (ns)	O: NO (40.13) vs. PO (63.54): *p* < 0.001 [0.001]
	O: *F*_(1.92,∞)_ = 42.63, *p* < 0.001		NO (40.13) vs. UO (25.59): *p* = 0.002 [0.002]
			PO (63.54) vs. UO (25.59): *p* < 0.001 [0.001]

M, music; O, odor; WN, white noise; PM, pleasant music; UN, unpleasant music; NO, no-odor; PO, pleasant odor; UO, unpleasant odor; FDR, false discovery rate (Bejamini and Hochberg, 1995); M_HD_, Harrell–Davis estimator of the median. The *p*-value of the FDR should be taken as the adjusted final *p*-value obtained.

Pairwise comparisons for the music factor failed to reach statistical significance. Although less conservative multiple comparisons tests can be used (and these do indeed reach statistical significance) the analyses are kept since they evidenced a statistically significant main effect of music on the pleasantness (*p* = 0.016), sweetness (*p* = 0.046), and dryness (*p* = 0.031) ratings. It is then possible to assert that the odors that were presented after white noise were rated as less pleasant, less sweet, and drier, as compared to the other conditions.

**FIGURE 3:**
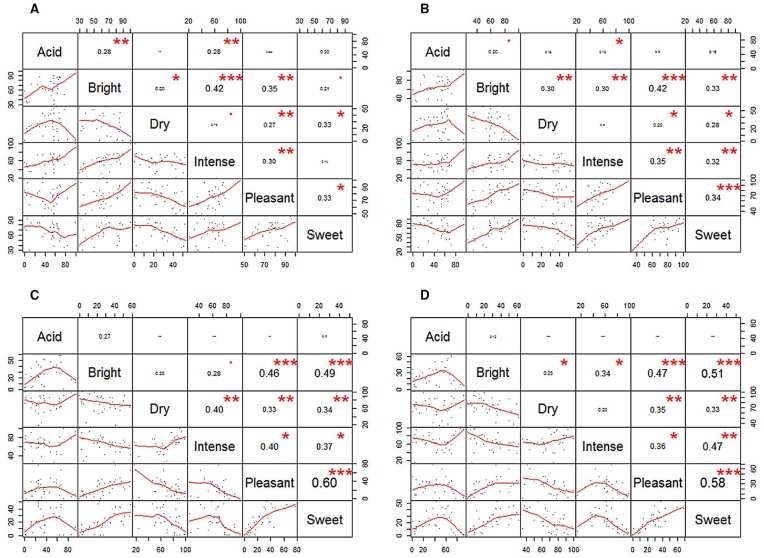
**Correlations between the ratings for the attributes of the pleasant music and pleasant odors (A), the white noise and pleasant odors (B), the unpleasant music and unpleasant odors (C), and the white noise and unpleasant odors (D) conditions.** The value within each box represents the Kendall tau coefficient and the *p*-values should be interpreted as follows: •*p* < 0.5, *p < 0.05, **p < 0.01, ***p < 0.001. In order to be coherent with the non-parametric tests used, locally weighted scatterplot smoothing (LOESS) lines (a non-parametric regression method) are shown instead of the traditional parametric straight lines (parametric regression method). Note, however, that interpretation of these lines is different from traditional ones. For example, the fitting lines in the scatterplot of intense-sweet **(D)** seem to indicate that there is a negative quadratic relationship, but this curvature is caused by only a few data points. The same occurs with intense-pleasant negative quadratic relationship.

In addition, Kendall tau correlations were performed between the different scores for each attribute for pleasant odors in the pleasant music and white noise conditions, and for unpleasant odors, in the unpleasant music and white noise conditions (see Figure 3 for a summary of the results).

Independently of the music condition, positive correlations were observed between brightness and pleasantness, brightness and sweetness, and pleasantness and sweetness. Moreover, a positive correlation was also documented between intensity and pleasantness in the pleasant music and white noise conditions for the pleasant odors, while a negative correlation was found between these attributes in the unpleasant music and white noise conditions, for the unpleasant odors. A positive correlation was found between acidity and brightness in all conditions except for the unpleasant music and unpleasant odors condition. In addition, a negative correlation was found between intensity and sweetness in the unpleasant music and white noise conditions for unpleasant odors, while a positive correlation between these attributes was found in the white noise condition for the pleasant odor. Taken together, then, these results suggest that although certain relationships seem to be unaffected by the music condition (e.g., the relationship between pleasantness and sweetness), certain others may change as a function of the condition (e.g., the relationship between intensity and pleasantness).

## DISCUSSION

The main aim of the present study was to assess whether musical pieces rated as pleasant or unpleasant would influence odors rated as either pleasant or unpleasant. Interestingly, when white noise was played in the background, a significant effect on participants’ ratings of the odor (relative to the other music conditions) was observed. When the odors were presented together with the white noise they were rated as less pleasant, as less sweet, and drier than in the other auditory conditions. This result provides evidence for the idea that white noise can have an effect on odor perception (when compared to a condition in which background music was played) and in particular it can influence participants’ hedonic ratings and perceived odor quality (e.g., sweetness).

Although, to the best of our knowledge, little previous research has been conducted to assess the influence of white noise on olfactory perception, suggestive evidence from its influence on the other senses may shed some light on its effects in the present study. Ferber and Cabanac (1987), for instance, conducted a study in the taste domain in which the hedonic valence of sucrose (but not of sodium chloride) solutions were shown to increase (meaning that people reported liking the solutions more) when listening to either loud noise or music. The sweet solutions were rated as significantly more pleasant when the participants were in the presence of the loud background noise or music (both presented at 90 dB over headphones) than when tasting in silence or while listening to quiet music (70 dB) instead. Interestingly, despite the fact that each person was allowed to listen to the music that they liked (that is, they were encouraged to bring their own preferred music into the study), it was the presumably unpleasant white noise that actually gave rise to the largest sweetness enhancement effects. Ferber and Cabanac (1987) suggested that this particular crossmodal effect may have been mediated indirectly via the modulatory effect of noise on participants’ arousal/stress levels which, in turn, may have affected their taste perception. It is important to note that this study only used 10 participants.

On the other hand, Masuda et al. (2008) reported that white noise had an effect on people’s ratings of the perceived moistness of pretzels. In addition, and more relevant to the aims of the present study, Woods et al. (2011) conducted two experiments designed to assess the effect of background noise on food perception and found that the perceived sweetness and saltiness of a variety of different foodstuffs, were lower in the white noise condition as compared to the other sound condition. Woods et al. (2011) concluded that background noise reduced the perception of gustatory attributes (see also Spence et al., 2014). It may be the case, then, that background white noise, or the hedonic value associated with it, not only affects the perception of gustatory but also olfactory attributes (Spence et al., 2014).

It is worth noting that white noise has been used as a neutral sound condition in previous studies on odor–sound interactions (Seo and Hummel, 2011; Seo et al., 2014). It may be the case that context can play an important role in guiding participants’ ratings (c.f., Martino and Marks, 2001); in other words, the combination of auditory stimuli that are used in the very same experiment may influence the way in which participants evaluate the different odors. That said, although people perceived the consonant and dissonant musical selections as respectively pleasant and unpleasant in the pre-test, the relative hedonic value of each musical piece may have changed when also including white noise (as shown in the follow-up experiment). This in turn, may have been reflected in the way in which white noise affected odor perception. Moreover, it may be the case that the consonant and dissonant pieces utilized were not sufficient to evoke a distinctive hedonic response, as compared to white noise (more salient).

In a follow-up study, we had participants rate the consonant and dissonant musical pieces on a hedonic scale (as done in the preliminary experiment) but in this case white noise was included, in order to assess whether participants’ ratings would be the same. The sounds were presented randomly four times to a group of 20 participants (eight females, *M*_age_ = 36.5 years, SD_age_ = 9.9, range = 23–55 years) who rated both their intensity and pleasantness on VAS scales anchored with *not at all* and *very much*. A repeated measures ANOVA (Greenhouse–Geisser-corrected) indicated a significant difference between the ratings, *F*_(2.248,42.721)_ = 53.751, *p* < 0.001, ${\text{η}}_{\text{p}}^{\text{2}}$ = 0.739. Pairwise comparisons using the Bonferroni correction revealed that participants rated white noise as significantly more unpleasant as compared to any of the other musical pieces (*p* < 0.001, for all comparisons). Importantly, no significant difference between the intensity ratings was found. Note that these results contrast with the preliminary experiment in which a difference was found between consonant and dissonant sounds, though white noise was not included. The aforementioned results point to the fact that the context created by particular sets of stimuli can influence participants’ ratings of those stimuli, which is consistent with the idea that context plays a key role in crossmodal correspondences.

Why should music and background sounds influence the perception of olfactory stimuli? As mentioned before, one possibility here is that the hedonic value (Kenneth, 1923; Marks, 1978; Deroy et al., 2013) of a stimulus presented to one sensory channel extends to the information presented in another (e.g., “halo effect,” see Seo and Hummel, 2011; Spence and Piqueras-Fiszman, 2014). Another possibility is that the information presented to one sensory modality captures more attention and thus influences the perception of the information in another modality (e.g., Boyle et al., 2006; Woods et al., 2011). These accounts are not necessarily incompatible in that participants may attend to information in one modality and if it is hedonically congruent with the other modality being evaluated, this may then strengthen its hedonic value. If it is not congruent (neither semantically nor hedonically), it may then interfere. Notably, this research is at an early stage, and thus further research is still needed in order to clarify the mechanisms underlying such phenomena.

Elsewhere, Spence and Shankar (2010) conducted a study in which they found that people’s rating of chocolate samples was unaffected by listening to consonant vs. dissonant triads. It is also plausible that under certain circumstances, particular consonant and dissonant sounds may not influence participants’ perception of odors/flavors (though see Sammler et al., 2007 for a study on how music can influence emotion).

Our results also contribute to the existing body of research suggesting that there is a relationship between pleasantness and odor qualities (e.g., Dravnieks et al., 1984; Stevenson and Boakes, 2004). That is, unpleasant odors were rated as dryer and more intense, as compared to the other odors, while pleasant odors were rated as sweeter and brighter, as compared to the others (see Zarzo and Stanton, 2009, for opposite uses of the descriptors sweet and dry when describing odors). Although the results presented here are limited in that only three odors per condition were included, they may nevertheless shed lights on how different olfactory percepts can be associated to the hedonic properties of odors. For instance, our results are in line with previous research (see Henion, 1971; Distel et al., 1999 for examples) suggesting that unpleasant odors are perceived as more intense (though matched in terms of their intensity in the pre-test); however, the association between pleasantness and intensity can be complex, and can vary as a function of the odors (e.g., Rouby et al., 2009); in other words, pleasantness and intensity can vary together and the direction of this variation depends upon the nature of the stimuli used, and the perceiver. On the other hand, previous research has suggested that people generally tend to prefer sweet smells (i.e., the smell of caramel, strawberry, vanilla, etc.; Stevenson and Boakes, 2004), which is also reflected in our results.

Notably, although our results contribute to the growing literature on the crossmodal influences in odor perception, a number of limitations should be mentioned. First, the main experiment did not include a no-sound condition, which limits the results to white noise as compared to dissonant and consonant sounds. Moreover, since participants were not explicitly informed about how to interpret each of the attributes used in this study (e.g., bright, dry), it is possible that their interpretation was not the same (e.g., Kaeppler and Mueller, 2013; though see Koulakov et al., 2011). In addition, although it has been documented that white noise can influence olfactory perception and that this may be attributed to its hedonic value (as compared to consonant and dissonant sounds), it is still an open question as to whether these effects are a product of some kind of “halo effect,” an attentional shift, or a combination of both. Future research may help to address this element and to extend the results to other food-related and non-food odors.

As a final note, it is worth mentioning that suggestive evidence from Wesson and Wilson (2010) indicates that the rat’s olfactory tubercle responds to tones, and that certain units in this structure show superadditive or suppressive responses when tones and odors are presented at the same time. Interestingly, animal studies suggest that there may be a strong evolutionary relationship between olfaction and audition (e.g., Cohen et al., 2011). This has been explained in terms of the idea that in natural environments animals may experience both olfactory and auditory stimuli at the same time in a variety of situations (e.g., think, for example, of the noise that can be heard when opening a fruit, while its smell is released). While the neural mechanisms that underlie olfactory–auditory interactions in humans remain largely unknown, scientists are now starting to point to the fact that auditory–olfactory crossmodal integration can potentially serve a variety of heretofore little considered functions (e.g., social perception of action, see Aglioti and Pazzaglia, 2011).

### Conflict of Interest Statement

The authors declare that the research was conducted in the absence of any commercial or financial relationships that could be construed as a potential conflict of interest.

## Footnotes

1 Note that effect sizes have not yet been proposed for this type of ANOVAs.
